# EFFECTIVENESS OF INTERVENTIONS FOR CHRONIC DISEASE MANAGEMENT AMONG OLDER ADULTS IN THAILAND: A META-ANALYSIS

**DOI:** 10.1093/geroni/igae098.3959

**Published:** 2024-12-31

**Authors:** Noppawan Piaseu, Phatcharaphon Whaikid

**Affiliations:** Mahidol University, Bangkok, Krung Thep, Thailand; Mahidol University, Bangkok, Krung Thep, Thailand

## Abstract

This study aims to assess effectiveness of interventions in managing chronic diseases in older Thais. We searched PubMed, Embase, Scopus, and Thai-Journal Citation Index Centre databases. This systematic review included articles published between 2018 and 2023, focusing on randomized controlled trials or quasi-experimental designs. Research quality was assessed using JBI critical appraisal tools. Meta-analysis employed JBI-SUMARI software. The protocol was registered on PROSPERO under identification code CRD42023475031. Thirty-seven articles met inclusion criteria, showing a significant effect favoring interventions. Systolic blood pressure (SBP) decreased by -12.27 mmHg (MD), 95% CI: -16.06, -8.47), and diastolic blood pressure (DBP) by -7.53 mmHg (95% CI: -9.72, -5.34). Subgroup analyses indicated a significant decrease in SBP due to self-management (MD -16.79 mmHg, 95% CI: -25.21, -8.38), lifestyle modification (MD -16.40 mmHg, 95% CI: -22.24, -10.56), physical and exercise (MD -8.77 mmHg, 95% CI: -13.02, -4.51), and application use (MD -3.49 mmHg, 95% CI: -5.73, -1.25). DBP decreased with self-management (MD -9.34 mmHg, 95% CI: -10.95, -7.72), lifestyle modification (MD -9.88 mmHg, 95% CI: -15.29, -4.47), physical and exercise (MD -4.65 mmHg, 95% CI: -6.51, -2.78), and application use (MD -4.09 mmHg, 95% CI: -5.92, -2.25). T2DM management decreased fasting blood sugar (SMD -0.78, 95% CI: -1.27, -0.311) and HbA1c (SMD -0.56, 95% CI: -0.81, -0.311). Lifestyle modification (SMD -0.70, 95% CI: -1.17, -0.24) and physical and exercise (SMD -0.46, 95% CI: -0.80, -0.13) decreased HbA1c. Self-management is effective in reducing BP in older Thais with hypertension, while lifestyle modification proves optimal for those with T2DM.

